# A bioinformatics analysis of the target role of miRNA-431-5p on KLK6 in colorectal cancer

**DOI:** 10.1186/s41065-025-00395-7

**Published:** 2025-03-28

**Authors:** Juan Wang, Zonglang Lai, Na Liu, Yuhong Wang, Feng Li, Na Song, Jun Cheng

**Affiliations:** Department of Oncology, Chongqing Traditional Chinese Medicine Hospital, Chongqing, 400021 China

**Keywords:** Colorectal cancer, WGCNA, KLK6, miR-431-5p, Bioinformatics

## Abstract

**Background:**

Although increasing evidence suggests that microRNAs (miRNAs) play different roles in the occurrence, development, and prognosis of colorectal cancer (CRC), investigations on miRNA-targeted regulation in CRC are sparse. However, the high morbidity and mortality of CRC necessitates exploring this area of research for potential alternative therapeutic approaches to CRC.

**Methods:**

Bioinformatics analysis was used to obtain the key Hub genes related to CRC, and survival analysis was performed to screen out the core genes. Meanwhile, verification was performed using CCK-8, Transwell, qPCR, WB, immunohistochemistry and dual luciferase assays at a cellular level.

**Results:**

This study identified the hub gene KLK6 associated with CRC based on the GEO and TCGA databases. Survival analysis revealed a significant decrease in the survival rate of CRC patients with increasing expression levels of KLK6. Target gene prediction confirmed that miR-431-5p can target KLK6. Cell experimental results demonstrated that the miR-431-5p inhibitor enhanced cell viability and promoted cell migration and invasion while miR-431-5p mimics reduced cell viability and inhibited cell migration and invasion. Both the inhibitor and mimics of miR-431-5p suppressed and promoted the expression of miR-431-5p, as well as promoted and inhibited the KLK6 mRNA and protein expression. Dual luciferase results showed that miR-431-5p targeted KLK6, and cell recovery experiments further verified that miR-431-5p regulated cell viability, migration and invasion by targeting KLK6.

**Conclusions:**

Through target gene prediction, miR-431-5p was found to target KLK6, suggesting its therapeutic potential in CRC. As such, therapies that can inhibit KLK6 via miR-431-5p offer a promising approach to CRC.

**Clinical trial number:**

Not applicable.

## Introduction

Colorectal cancer (CRC) is one of the most common gastrointestinal malignancies in humans, the third most common tumor and the fourth leading cause of cancer-related mortality worldwide [1]. According to global cancer statistics [2], in 2020, there were 1.9 million new patients in the world, accounting for 10% of the cancer incidence; 0.93 million new deaths, with a mortality rate of 9.4%, and morbidity and mortality showed an increasing trend year by year. Among them, there were 555,477 new CRC patients in China, accounting for 12.2% of the total incidence of cancer [3]. CRC also ranks second in the country, with 286,162 new deaths and a mortality rate of 9.5%, ranking fifth in the country [4]. Although the diagnosis and treatment of CRC are improving, the prognosis of advanced patients remains highly unfavorable, with a relatively high mortality rate and a low five-year survival rate [5, 6]. Tumor markers play an important role in the early diagnosis, prognosis and efficacy evaluation of tumors [7, 8]. Therefore, finding new therapeutic targets and exploring their mechanisms are vital to guide the clinical CRC treatment.

MicroRNA (miRNA) is a type of single-stranded noncoding RNA ranging between 21 and 25 nucleotides in length, which can cause translation inhibition or degradation of target messenger RNA (mRNA) by specifically binding to it, thereby regulating gene expression [9]. In addition, miRNA is also involved in regulating cell development, differentiation, proliferation, apoptosis, immune response, angiogenesis, and other processes [10]. MiRNAs play an emerging role in controlling key pathways related to tumor development, making them a powerful new tool for early detection, risk assessment, prognosis, and the development of innovative anti-cancer therapies [11]. Increasing evidence suggests that miRNAs are involved in the occurrence, development, and prognosis of cancers such as hepatocellular carcinoma [12], tongue squamous cell carcinoma [13] and CRC [14]. Michael et al. [15] first reported studies on miRNA expression in colorectal tumor tissue, showing downregulation of miR-143 and miR-145 in tumor tissues. Mazeh et al. [16] have further found that 170 different miRNAs are upregulated and 127 different miRNAs are downregulated in CRC. For example, the expression of miR-J1-5p decreases with the progression of CRC [17], and microRNA (miR)-216b levels are significantly downregulated in CRC cells treated with anti-EGFR therapy [18]. Furthermore, miRNAs can target genes to regulate tumor development, such as MicroRNA-431-5p inhibiting osteosarcoma tumorigenesis by targeting PANX3 [19]. However, there is currently limited research on miRNA-targeted regulation in CRC. Using a bioinformatics approach, this study seeks to investigate the target role that miRNA-431-5p exerts on KLK6 in CRC.

Tools based on gene chips combined with bioinformatics have recently emerged and have been widely used to study the pathogenesis of complex diseases, including the identification of biomarkers and in predicting disease progression and prognosis [20, 21]. These tools also have diagnostic and prognostic potentials in various cancers [22–24]. In line with this genetic data-based approach to investigating CRC, the current study integrated CRC-related data from GEO and TCGA databases to obtain key Hub genes associated with CRC by bioinformatics analysis. The survival analysis of selected key Hub genes was conducted by using the online tool GEPIA and subsequently the possible relationship between the hub genes and CRC prognosis was investigated. Meanwhile, the core genes related to prognosis were selected, and the expression of which related to prognosis was determined. Through bioinformatics predictions and cellular experiments validation, potential therapeutic targets for CRC were identified and their molecular mechanisms were explored.

## Materials and methods

### Screening of differentially expressed miRNA and mRNA

MiRNA data sets GSE130084 were obtained from a GEO database (https://www.ncbi.nlm.nih.gov/geo/), including 2 normal samples and 2 CRC samples; 9 healthy and 10 CRC samples were downloaded from TCGA database. Differentially expressed mRNAs were analyzed through using edgeR software in R package and differentially expressed miRNA were analyzed through using DESeq2 software in R package, with p values less than 0.05 and|log_2_FC| more than 1. The clustering heatmap and volcano map were plotted and analyzed with the pheatmap software package and ggplot2 software package in R software.

### Pathway enrichment analysis

Enrichment analysis involves comparing the target gene set with the gene distribution in the whole-genome background to determine whether these genes are significantly concentrated in certain specific functional categories or biological pathways. DAVID database was used for enrichment analysis of gene ontology function (GO) and the Kyoto Encyclopedia of Genes and Genomes (KEGG), and the enrichment results were visualized with R software set at *p* < 0.05 and FDR < 0.05 conditions.

### Weighted gene co-expression network analysis (WGCNA)

Co-expression network was constructed by using WGCNA package in R language.

The samples were firstly clustered with outlier samples deleted. Secondly, a scale-free network was constructed by screening the soft threshold β. In this process, the Pearson correlation coefficient was used to calculate the gene similarity based on the gene expression data, and a similarity matrix was constructed. Subsequently, the soft threshold β was screened to make the network degree distribution conform to the power-law distribution. Then, the similarity matrix was processed with the soft threshold to obtain the adjacency matrix. Based on this, the topological overlap matrix (TOM) was calculated. Finally, genes with similar expression patterns were divided into one module using a dynamic shear tree.

### Construction of protein-protein interaction (PPI) network map and screening of hub genes

An online tool STRING (https://string-db.org/) was applied to construct PPI network maps. MCC algorithm in cytoHubba plugin of Cytoscape3.8.0 was used to select top 10 genes in PPI network as hub genes. These genes are the most central ones, having the largest number of connection relationships. Selecting the top 10 genes can not only ensure the representativeness and operability of the results, but also avoid data complexity. This number is sufficient for in-depth analysis and endows the results with biological significance.

### Expression verification and survival analysis of key genes

The mRNA expressions of core genes in CRC samples and normal samples were verified and visualized using GEPIA database. GEPIA was used to evaluate the relationship between hub gene expression and overall survival (OS) in CRC patients, and to screen the core genes associated with prognosis.

### Cell treatment and grouping

HCT116 cells were purchased from Wuhan Pricella Biotechnology Co., Ltd. (CL-0096, Wuhan, China). Cell suspension was prepared from well-grown cells. 10 µL of cell suspension was mixed with 10 µL of 0.4% trypan blue for staining; blood cells were counted, and cell density was adjusted to 2 × 10^5^/mL, and then the cell suspension was added to 6-well plates with 2 mL of culture solution for culture. After the cells were adhered to the wall, the solution was replaced using a complete culture medium, which was used for transfection of inhibitor empty-load, miR-431-5p inhibitor, mimics empty-load, and miR-431-5p mimics respectively according to the grouping for 48 h for subsequent determination.

### Cell rescue assay

HCT116 cells were planted in a culture dish at a density of 5 × 10^6^ per dish and fresh MEM culture medium was used after 24 h. Four 1.5 mL EP tubes were prepared, and 10 µg of each mimic and 10 µg of plasmids were added to one of the EP tubes according to the grouping (mimics-NC + oe-NC, miR-431-5p mimics + oe-NC, mimics-NC + oe-KLK6, miR-431-5p mimics + oe-KLK6), dissolved in 490 µL of OPTI-MEM, gently mixed, and incubated at room temperature. A solution containing transfection reagent was added, mixed well, let undisturbed at room temperature for 15 min, and then evenly added to the culture dishes. The agents were mixed thoroughly using a cross method, and incubated in the cell culture incubator for 24 h before sample collection for subsequent analyses.

### CCK-8

In a 96-well plate containing cells, 100 µL of CCK-8 solution (C0038, Beyotime, China) was added to each well with a mixture of complete medium (1:10), and the absorbance was measured at 450 nm using a microplate reader (CMax Plus, molecular devices, USA) after 1 h incubation in a cell incubator.

### Transwell detection of cell migration

The cells were cultured under good conditions, and resuspended with a serum-free medium, and the cell density was adjusted to 5 × 10^5^/mL. Cell suspension at 100 µL was added to each well in the Transwell chambers (3421, Corning) and incubated in a 5% incubator at 37 °C for 24 h. The cells were fixed with 4% paraformaldehyde, stained with 0.1% crystalline violet (1425163, Leagene, China), observed under a microscope and counted.

### Transwell detection of cellular invasion

After mixing the serum-free medium and Matrigel (356234, Corning, USA) (5:1), 100 µL was added to the upper Transwell chamber and incubated at 37 °C for 4 ~ 5 h. After the digestion was discontinued, the cells were resuspended in a serum-free medium and the cell density was adjusted to 5 × 10^5^/mL. The coagulated Matrigel was washed with the serum-free medium and added with 100 µL of cell suspension per well. After the addition of 500 µL of conditioned medium containing 20% FBS to the lower chamber, the cells were incubated at 37 °C in a 5% incubator for 24 h. Following fixation with 4% paraformaldehyde, the cells were stained using a crystal violet staining, visualized random fields under a microscope and counted.

### qPCR

Cellular RNA was extracted using TRIzol reagent; cDNA synthesis was performed using Goldenstar™ RT6 cDNA Synthesis Kit Ver.2 (TSK302M, Tsingke, China), and qRT-PCR was performed according to 2 × T5 Fast qPCR kit instructions (TSE002, Tsingke, China). The housekeeping gene GAPDH was used for normalization, and primer sequences are shown in Table 1.

**Table 1:** Primer sequences

Primers	Sequence
miR-4732-5p stem loop	CTCAACTGGTGTCGTGGAGTCGGCAATTCAGTTGAGCTGCATGAC
h-miR-431-5p-F	TCGGCAGGTGTCTTGCAGGCCG
h-miR-431-5p-R	CTCAACTGGTGTCGTGGA
h-U6-F	CTCGCTTCGGCAGCACA
h-U6-R	AACGCTTCACGAATTTGCGT
h-KLK6-F	ACAGAACCAGCCTCTTCCAG
h-KLK6-R	AGTTAGAAATGCGGGGAGCC
h-GAPDH-F	TCAAGGCTGAGAACGGGAAG
h-GAPDH-R	TCGCCCCACTTGATTTTGGA

### Western blotting

Total protein was extracted from the sample using RIPA lysate (P0013B, Beyotime, China) and its concentration was detected. 500 µg was used and mixed with a 5× SDS loading buffer (G2083, Servicebio, China) in a 4:1 ratio, and heated in a metal bath at 100 °C for 6 min for protein denaturation. Subsequently, 60 µg of the denatured total protein was loaded, sealed using a 5% skim milk blocking solution at room temperature for 1 h, and incubated with primary antibodies (KLK6 (1:1000, A12055, abclonal, China) and GAPDH (A12055, abclonal, China)) overnight at 4 °C. Secondary antibodies (1:2000, AS014, abclonal, China) were also supplied for incubation at room temperature for 1 h. The ECL exposure solution (34580, Thermo, USA) was mixed with Solution A: Solution B = 1:1 and evenly covered on the entire film. Following 1 min reaction, an exposure instrument was applied for exposure detection.

### Dual luciferase reporter gene assay

The highest scoring binding site of miR-431-5p on the 3’UTR of KLK6 was selected to construct the luciferase reporter vector pmiR-3’UTR-WT-luc2-Rluc, and the binding site was mutated to construct pmiR-3’UTR-mut-luc2-Rluc. miR-431-5p mimics were synthesized and co-transfected with a dual luciferase reporter gene vector into 293T cells. Specifically, 50 µL of antibiotics and serum-free medium were added and 1 µg of plasmid DNA, aspirated and mixed evenly. Next, 1.6 µL of Nanofusion transfection reagent was supplied, mixed by aspiration again, let stand at room temperature for 5–20 min, and then added with the cells in a 12-well plate. The cells were divided into five groups: NC + pmiR-NC-luc2-Rluc, NC + pmiR-3’UTR-WT-luc2-Rluc, miR-mimics + pmiR-NC-luc2-Rluc, miR-mimics + pmiR-3’UTR-WT-luc2-Rluc, and miR-mimics + pmiR-3’UTR-mut-luc2-Rluc. After transfection, detection was performed according to the instructions for the luciferase assay kit (RG027, Beyotime, China).

### Immunohistochemistry

The fixed cell slides were washed with PBS, blocked at room temperature with goat serum (C0265, Beyotime, China) for 30 min and excess serum was removed. Primary antibody (1:200, KLK6, YC0182, Immunoway, Texas, USA) was added for incubation overnight at 4 °C. After PBS washing, the slides were incubated by added with secondary antibody (1:500, 511203, ZEN-BIOSCIENCE, Chengdu, China) working solution for 1.5 h at room temperature. Following PBS washing again, DAB color development was performed. The samples were counterstained with hematoxylin, differentiated in 1% hydrochloric acid ethanol, rinsed in tap water for 10 min, dehydrated with ethanol, transparanted with xylene, and sealed with neutral resin. Microscope observation indicated blue in color for cell nuclei, and brown-yellow for positive areas.

### Statistical analysis

All experiments were repeated three times using Graphpad Prism 7.0 software for statistical analyses and plotting. All results were expressed as mean ± standard deviation (X ± S), and independent sample *t*-tests were used for pairwise comparison. The value of *p* < 0.05 was considered to be statistically significant.

## Results

### Analysis of differentially expressed miRNAs and mRNAs

**Fig. 1:**
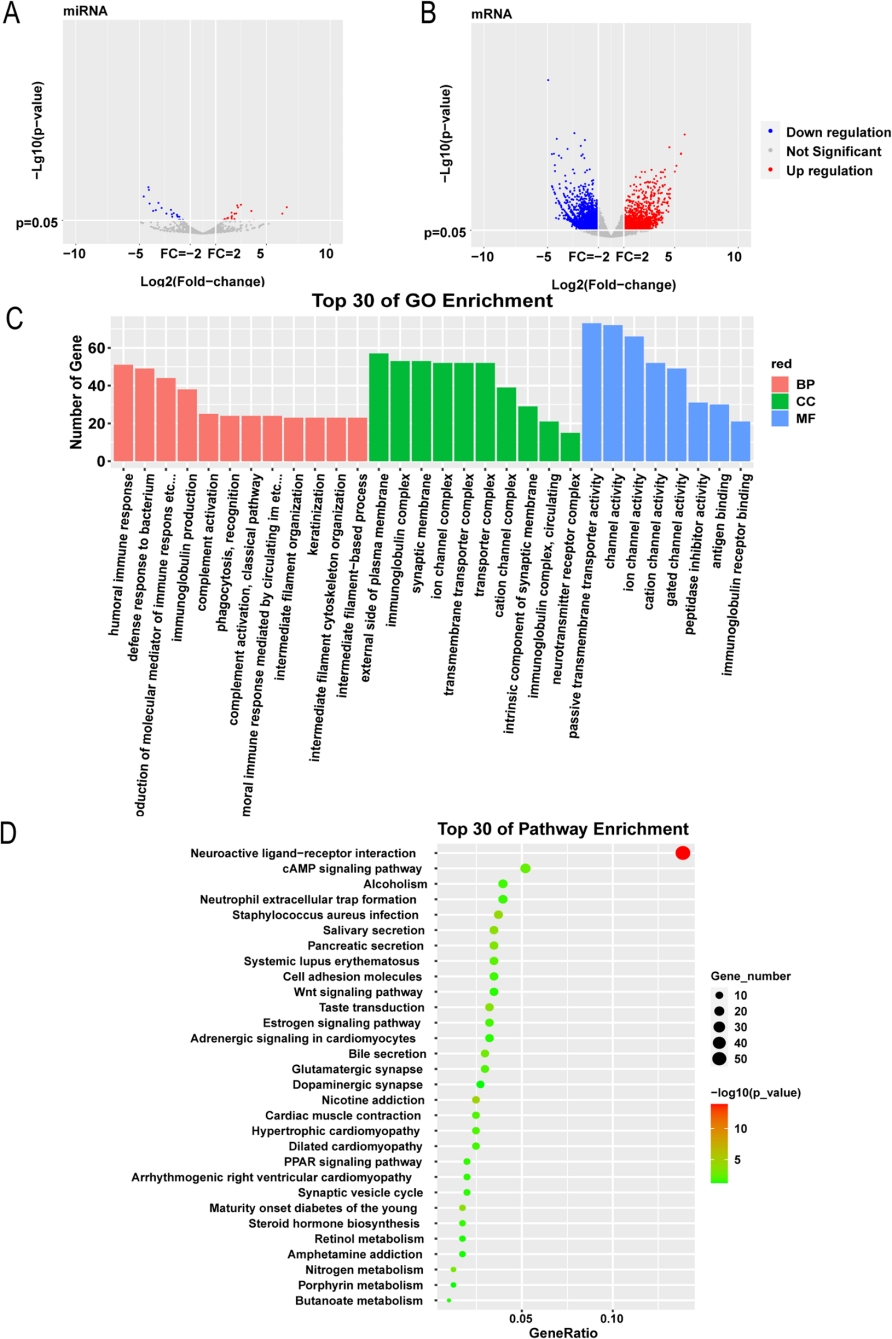
Analysis of Differentially Expressed miRNAs and mRNAs. (**A**) Differentially expressed miRNA screening. (**B**) Screening of differentially expressed mRNAs. (**C**) Bar graph of GO enrichment analysis. (**D**) Scatter plot of KEGG enrichment analysis

Based on the sample data in the data set GSE130084, with *p* values < 0.05 and|log_2_FC| ≥ 1 as the screening conditions, 39 significantly different miRNAs related to CRC were screened, of which 19 were up-regulated and 20 down-regulated (Fig. 1A).

Among the collected 3716 significantly differentially expressed mRNAs from TCGA data, 1839 of them were up-regulated and 1877 down-regulated (Fig. 1B).

GO enrichment analysis showed that DEGs were mainly enriched in humoral immune response, defense response to bacterium, and production of molecular mediator of immune response. in BP; the mostly enriched terms in CC included external side of plasma membrane, immunoglobulin complex, and synaptic membrane; those enriched in MF included ion channel activity, channel activity, and passive transmembrane transporter activity (Fig. 1C). KEGG enrichment indicated that the mostly enriched pathways of DEGs were cAMP signaling pathway, neuroactive ligand-receptor interaction, neutrophil extracellular trap formation and Wnt signaling pathway (Fig. 1D).

### WGCNA analysis of DEGs

**Fig. 2:**
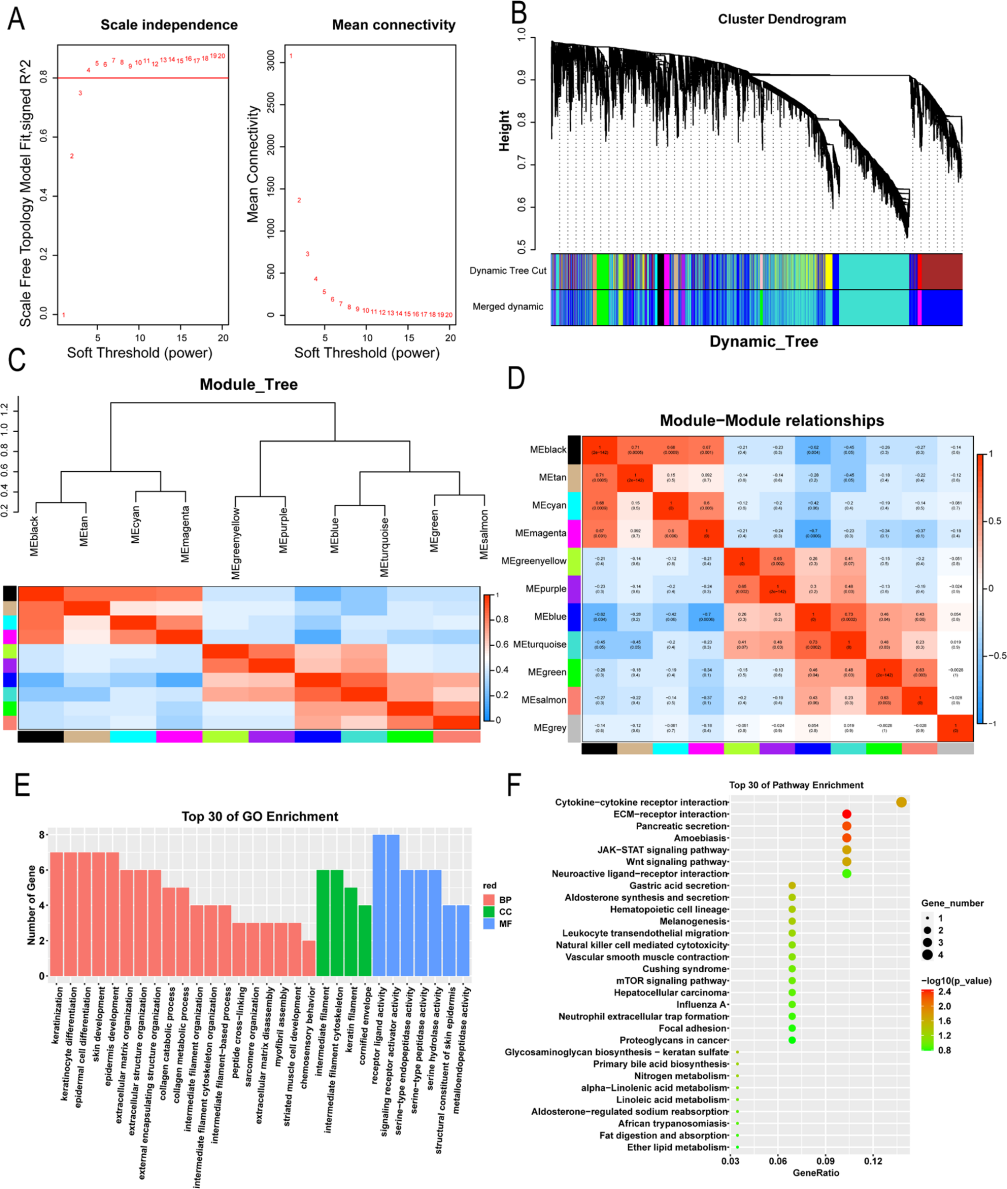
WGCNA and enrichment analysis. (**A**) Scale-free index analysis of various soft threshold powers (β). (**B**) Cluster module tree of DEGs (top) and color tape (bottom). (**C**) Gene clustering tree based on topological overlap, and assigned module colors. (**D**) Analysis of correlations between modules and disease traits. (**E**) Bar graph of GO enrichment analysis. (**F**) Scatter plot of KEGG enrichment analysis

The scale-free network was constructed by selecting soft threshold power β = 3 (R^2^ = 0.8) (Fig. 2A). This study identified 11 co-expression modules (Fig. 2B and C). The modular-clinical feature correlation heatmap was used to assess the correlation between the module and clinical features (tumor and normal) (Fig. 2D). Among these modules colored in black (*r* = 0.73, *p* = 0.0003), magenta (*r* = 0.7, *p* = 0.0006), blue (*r*=-0.96, *p* = 4e-11) and turquoise (*r*=-0.72, *p* = 0.0004) were strongly correlated with tumor tissue. Genes in the black and magenta modules were selected for subsequent analysis. GO enrichment results showed that module genes were mostly concentrated in the aspect of BP, such as keratinization, keratinocyte differentiation, and epidermal cell differentiation; In terms of CC, it was mainly concentrated in intermediate filtration, and intermediate filtration cytoskeleton; In MF, it was mainly signaling receptor activator activity, concentrated in receptor ligand activity, and serine-type endopeptidase activity (Fig. 2E). KEGG results showed that module gene enrichment focused on ECM-receptor interaction, cytokine-cytokine receptor interaction, JAK-STAT and Wnt signaling pathways (Fig. 2F).

### Hub gene screening and the prognosis of survival analysis

**Fig. 3:**
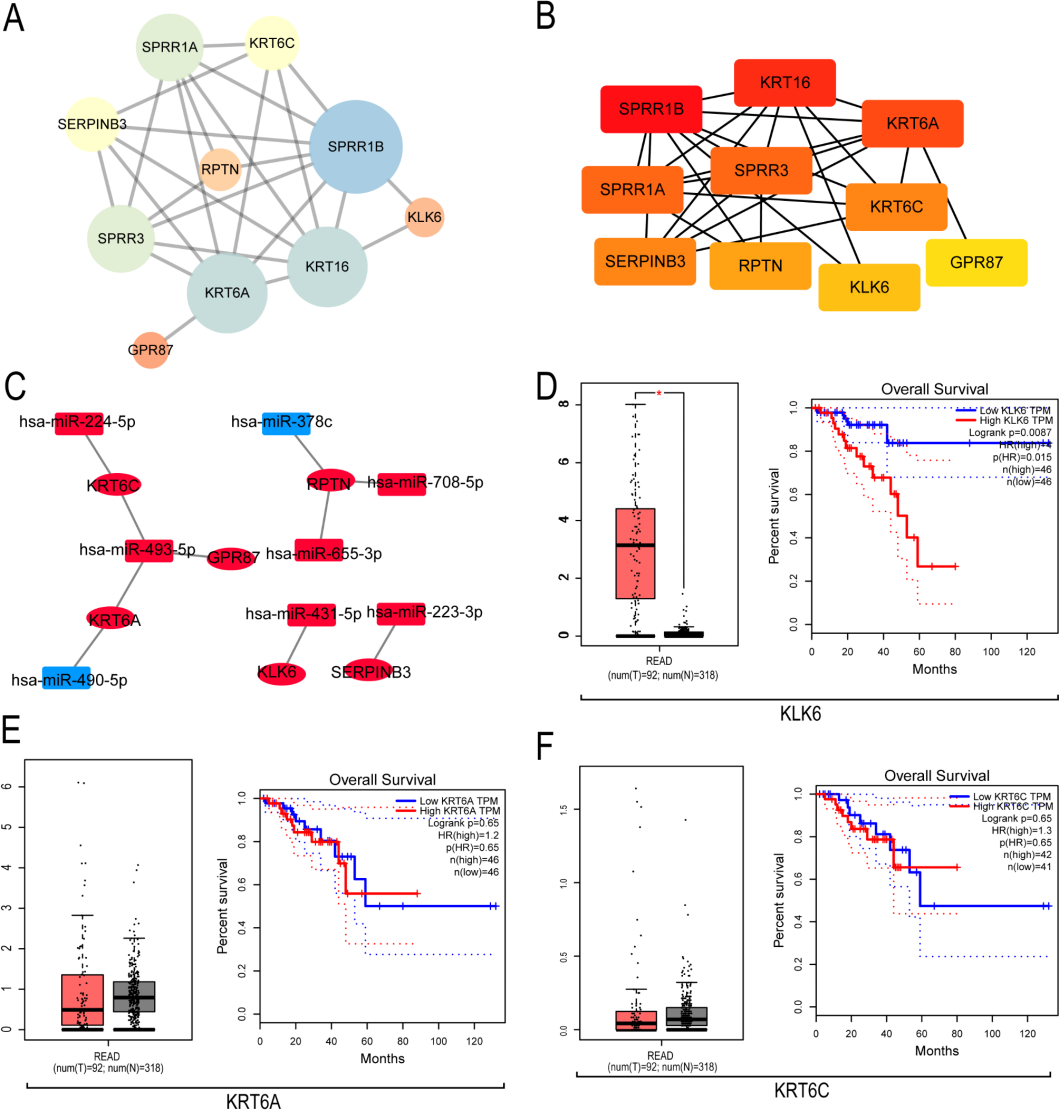
Hub genetic screening and the prognosis of survival analysis. (**A**) PPI Network of DEGs. (**B**) The Hub Genes of Its Core Module. (**C**) Targeting relationship prediction. (**D**) Prognostic analysis and expression verification of KLK6. (**E**) Prognostic analysis and expression verification of KRT6A. (**F**) Prognostic analysis and expression verification of KRT6C

PPI networks showed correlations between proteins in modules black and magenta (Fig. 3A). The described hub genes in PPI network included KRT16, SPRR1B, KRT6A, SPRR3, SPRR1A, KRT6C, SERPINB3, RPYN, KLK6 and GPR87 (Fig. 3B). Target gene prediction analysis showed that miR-493-5p could target KRT6C, KRT6A and GPR87; MiR-224-5p targeted KRT6C; MiR-490-5p targeted KRT6A; MiR-378c, miR-708-5p and miR-655-3p could target RPTN; MiR-431-5p targeted KLK6; MiR-223-3p targeted SERPINB3 (Fig. 3C).

Based on GEPIA database, the mRNA expressions of key genes KLK6, KRT6A and KRT6C in CRC samples and normal samples were verified, implying that KLK6 expression in tumor tissues was substantially elevated as compared to normal tissues (*p* < 0.05, Fig. 3D); KRT6A level in tumor tissues was increased as compared to normal tissues without significant difference (*p* > 0.05, Fig. 3E). Conversely, KRT6C expression in tumor tissues declined as compared to normal tissues without significant difference (*p* > 0.05, Fig. 3F). The survival curve visualizes the relationship between hub genes and survival prognosis in CRC patients. KLK6 gene intimately linked to OS (*p* < 0.05), and patients with high KLK6 expression had a worse prognosis than those poorly expressed (Fig. 3D). There was no significant correlation between KRT6A and KRT6C and patients’ OS (*p* > 0.05, Fig. 3E and F).

### miR-431-5p regulates the expression of KLK6

**Fig. 4:**
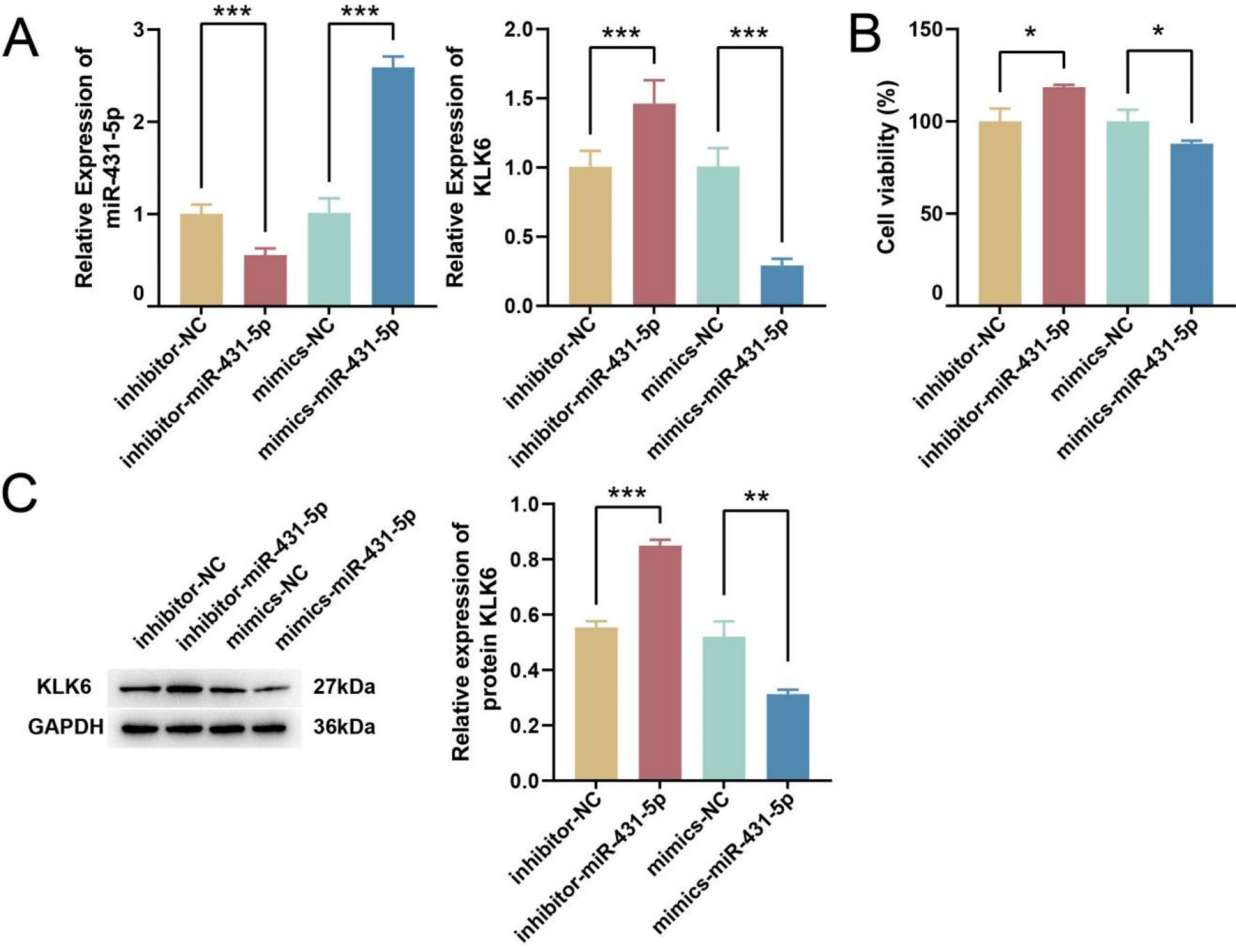
miR-431-5p targets KLK6 validation. (**A**) qPCR detection of expression of miR-431-5p and KLK6 after transfection. (**B**) CCK-8 detection of cell viability after transfection. (**C**) WB detection of expression of KLK6 protein after transfection. * *p* < 0.05, ** *p* < 0.01, *** *p* < 0.001

**Fig. 5:**
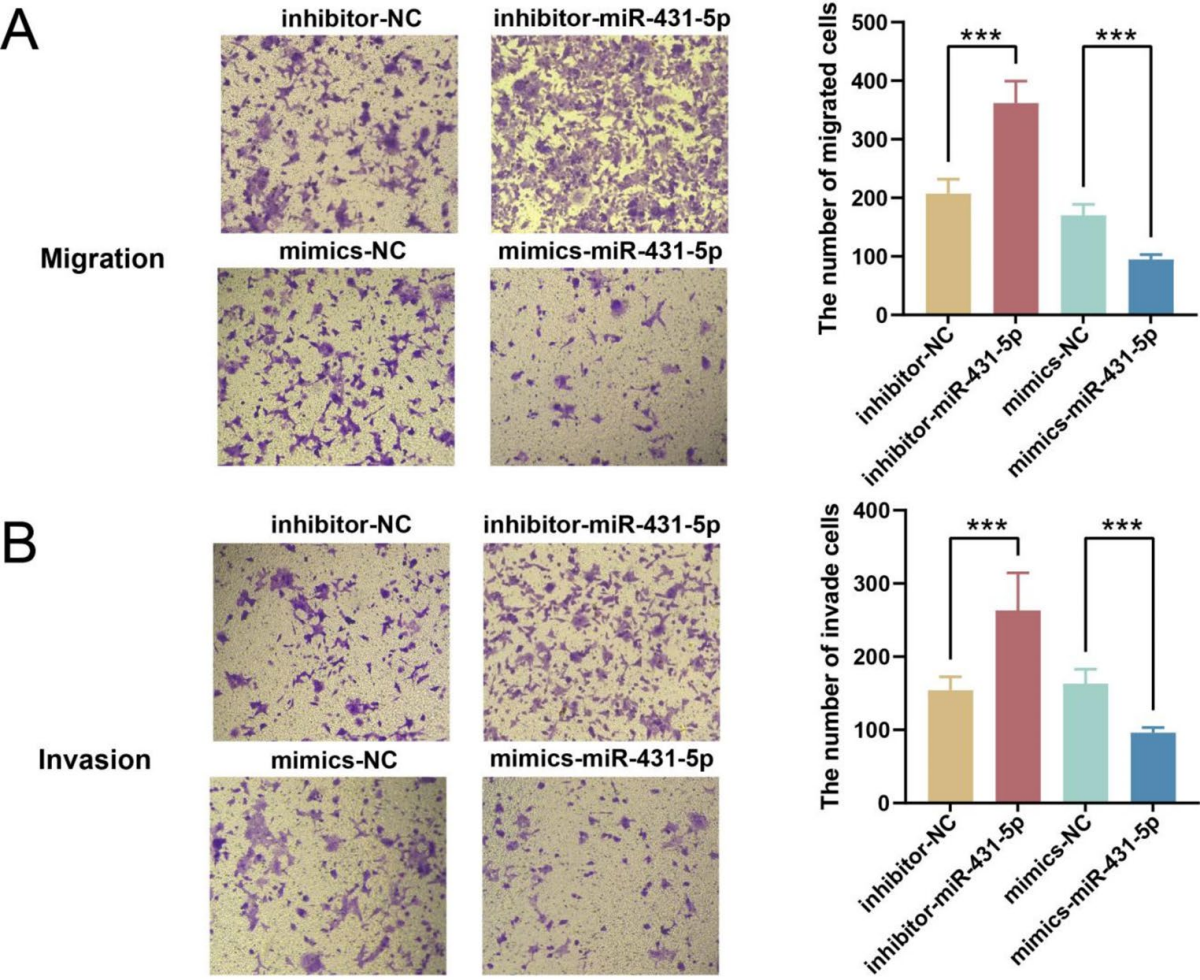
Transwell detection of HCT116 cell migration and invasion abilities (**A**) Transwell assay for cell migration after miR-431-5p transfection (100×). (**B**) Transwell assay for cellular invasion after miR-431-5p transfection (100×). *** *p* < 0.001

The PPI network analysis revealed that miR-431-5p targeted KLK6 (Fig. 3C), and survival prognosis analysis showed that patients with high expression of KLK6 had a poorer prognosis than those with low expression (Fig. 3D). To further verify the effect of miR-431-5p targeting KLK6, we transfected miR-431-5p inhibitor or mimics in HCT116 cell lines. The qPCR results showed that the inhibitor and mimics of miR-431-5p respectively inhibited and promoted the expression of miR-431-5p (*p* < 0.001, Fig. 4A), and the opposite for KLK6. The inhibitor and mimics of miR-431-5p significantly promoted and inhibited the expression of KLK6 mRNA (Fig. 4A). The inhibitor and mimics of miR-431-5p significantly increased and decreased cell viability, respectively (*p* < 0.05, Fig. 4B). The WB results showed that compared to inhibitor-NC, inhibitor-miR-431-5p significantly increased the expression of KLK6 protein (*p* < 0.001). In comparison to mimics-NC, mimics-miR-431-5p significantly decreased the expression of KLK6 protein (*p* < 0.01, Fig. 4C). miR-431-5p inhibitor significantly improved the migration and invasion ability of HCT116 (*p* < 0.001), and miR-431-5p mimics significantly reduced the migration and invasion ability (*p* < 0.001, Fig. 5A and B).

### miR-431-5p targets KLK6

**Fig. 6:**
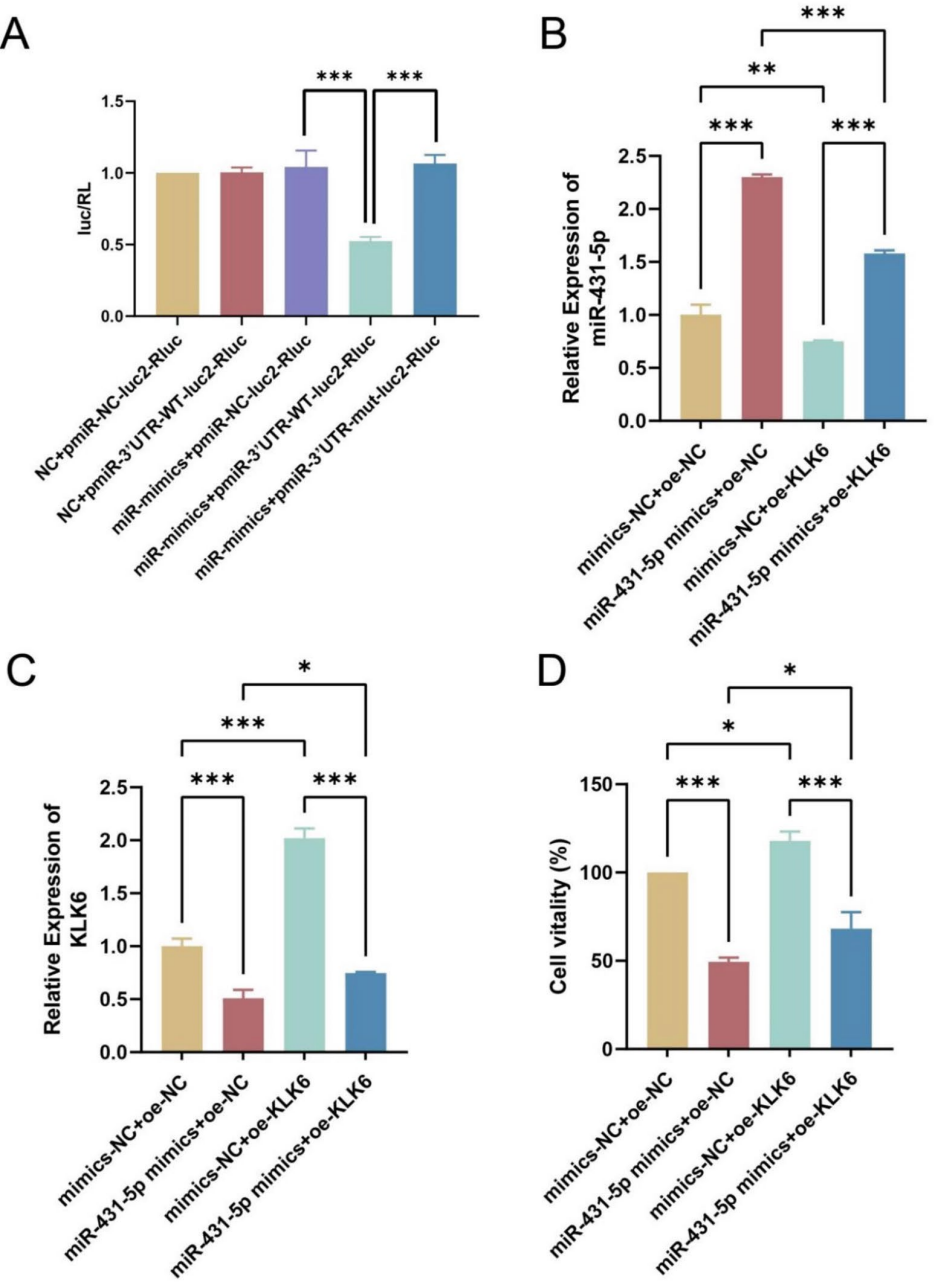
miR-431-5p targets KLK6. (**A**) Dual luciferase reporter gene assay was used to verify the relationship between miRNA and target gene; (**B**) The expression of miR-431-5p in HCT116 cells was detected by qPCR; (**C**) qPCR was used to detect the expression of KLK6 mRNA in HCT116 cells; (**D**) CCK-8 assay was performed to detect the cell proliferation of HCT116 cells. * *p* < 0.05, ** *p* < 0.01, *** *p* < 0.001

**Fig. 7:**
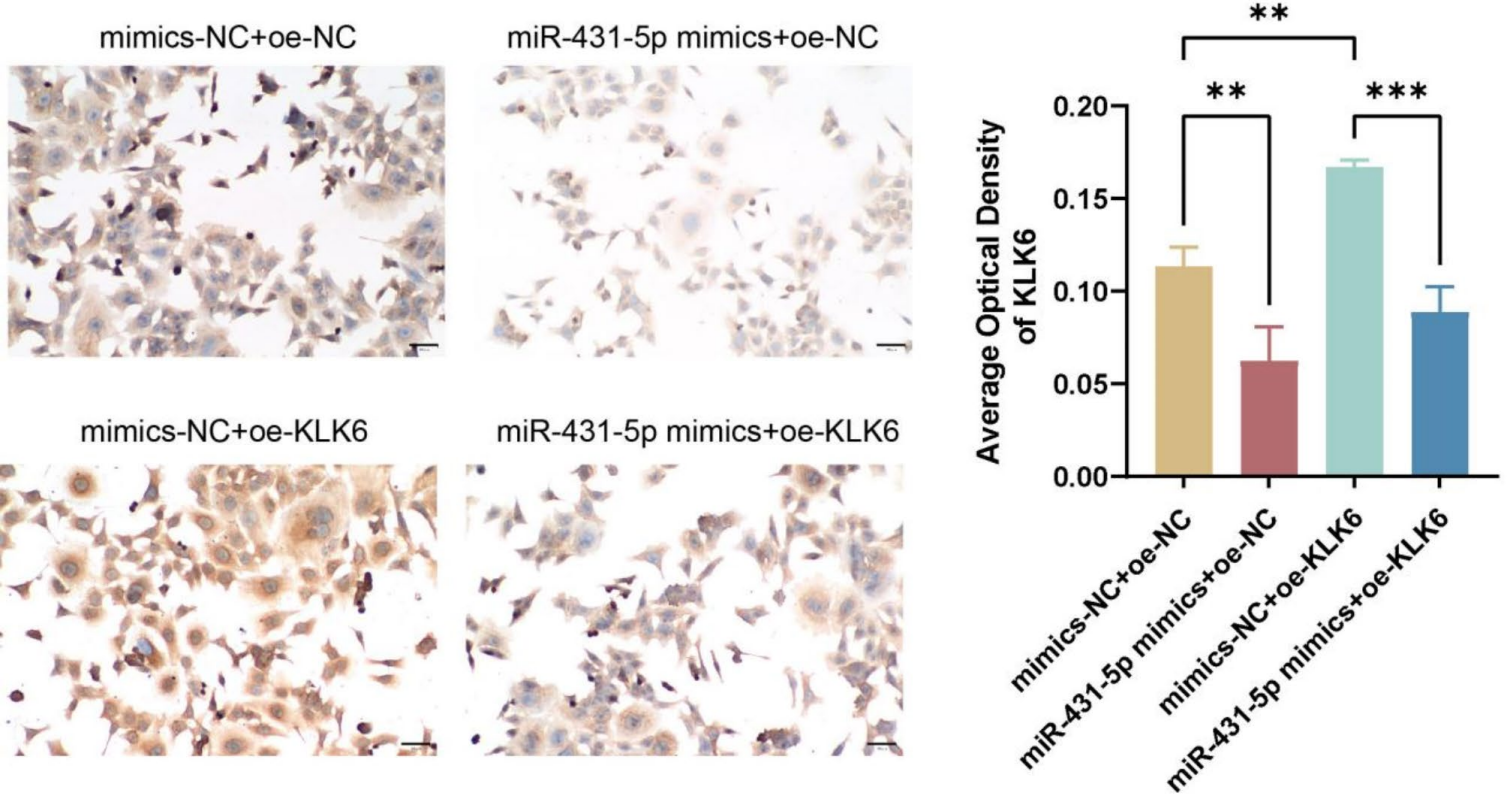
The expression of KLK6 protein in HCT116 cells is detected by immunohistochemistry. ** *p* < 0.01, *** *p* < 0.001

To further validate the relationship between miR-431-5p and KLK6, we conducted a dual-luciferase reporter gene assay. The results demonstrated that miR-431-5p mimics inhibited the luciferase activity of the KLK6 reporter plasmid, while there was no significant effect after mutation of the miR-431-5p binding site (Fig. 6A). Subsequently, cell rescue assays were conducted, and qPCR results indicated that compared to the mimics-NC + oe-NC group, miR-431-5p expression was significantly increased in the miR-431-5p mimics + oe-NC group but significantly decreased in the mimics-NC + oe-KLK6 group (*p* < 0.01, *p* < 0.001). However, the expression of miR-431-5p in the miR-431-5p mimics + oe-KLK6 group was significantly lower than that in the miR-431-5p mimics + oe-NC group but significantly higher than that in the mimics-NC + oe-KLK6 group (*p* < 0.001, Fig. 6B). Conversely, the expression of KLK6 exhibited an opposite trend (Fig. 6C). CCK-8 results showed that compared to the mimics-NC + oe-NC group, cell viability was significantly inhibited in the miR-431-5p mimics + oe-NC group but significantly promoted in the mimics-NC + oe-KLK6 group (*p* < 0.05, *p* < 0.001). Moreover, cell viability in the miR-431-5p mimics + oe-KLK6 group was significantly higher than that in the miR-431-5p mimics + oe-NC group and significantly lower than that in the mimics-NC + oe-KLK6 group (*p* < 0.05, *p* < 0.001, Fig. 6D). The immunohistochemistry results showed that compared to the mimics-NC + oe-NC group, the positive expression in the mimics-NC + oe-KLK6 group significantly increased (*p* < 0.01), while the positive expression in the miR-431-5p mimics + oe-NC group significantly decreased (*p* < 0.01). Compared to the mimics-NC + oe-KLK6 group, the positive expression in the miR-431-5p mimics + oe-KLK6 group significantly decreased (*p* < 0.001, Fig. 7). These results indicated that miR-431-5p could target KLK6.

### miR-431-5p targets KLK6 to regulate cells

**Fig. 8:**
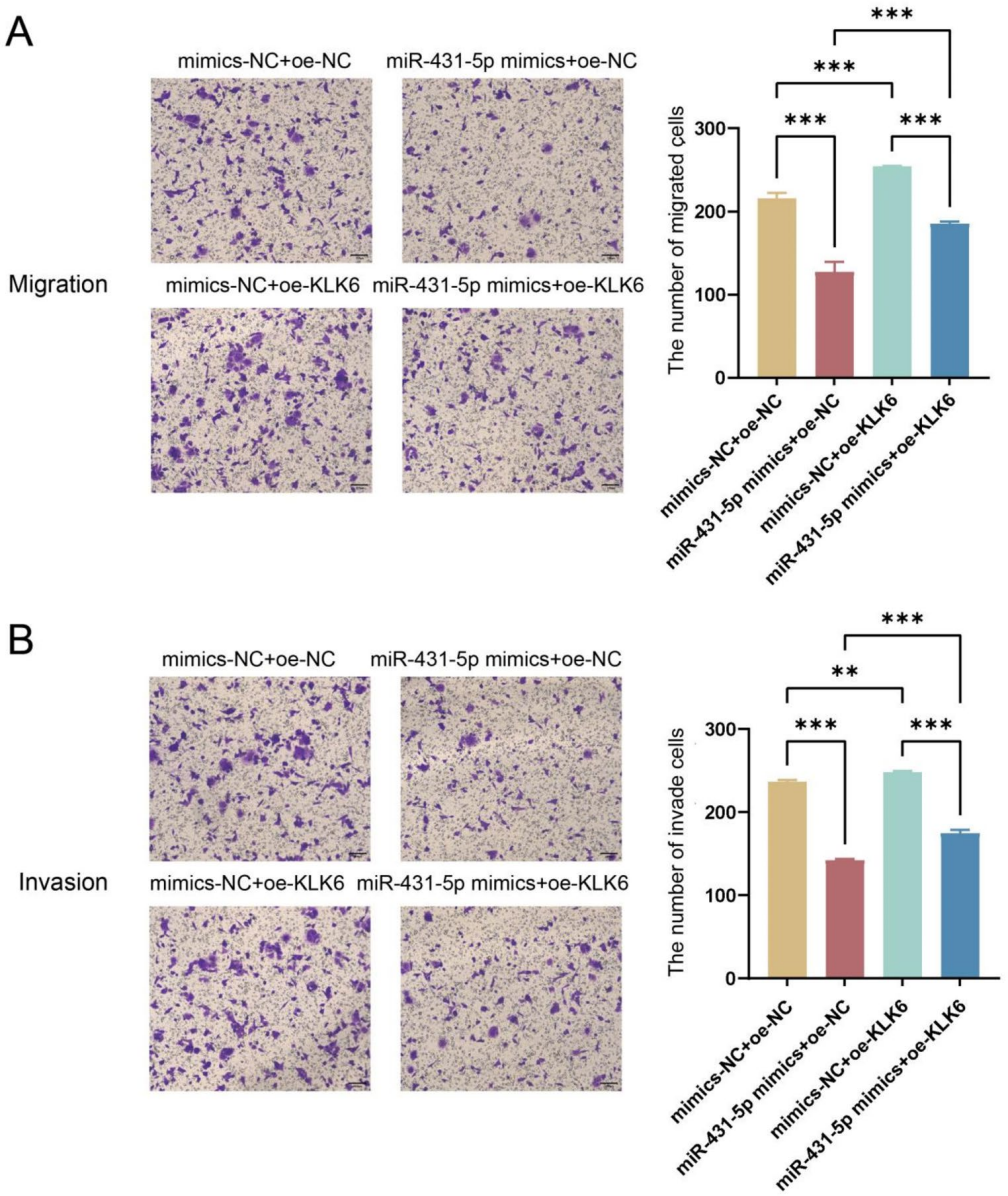
miR-431-5p targets KLK6 to regulate cell migration and invasion. (**A**) The migration ability of HCT116 cells was detected by Transwell (200×); (**B**) The invasion ability of HCT116 cells was measured by Transwell (200×). ** *p* < 0.01, *** *p* < 0.001

Transwell assay results (Fig. 5) showed that, compared to the mimics-NC + oe-NC group, the migration and invasion abilities of the miR-431-5p mimics + oe-NC group were significantly reduced (*p* < 0.001), consistent with previous results. In the mimics-NC + oe-KLK6 group, the migration and invasion abilities were significantly increased (*p* < 0.01, *p* < 0.001), indicating that overexpression of KLK6 could promote cell migration and invasion abilities. Compared to the miR-431-5p mimics + oe-NC group, the migration and invasion abilities in the miR-431-5p mimics + oe-KLK6 group were also significantly increased (*p* < 0.001). Compared to the mimics-NC + oe-KLK6 group, the miR-431-5p mimics + oe-KLK6 group exhibited significantly reduced migration and invasion abilities (*p* < 0.001, Fig. 8A and B), indicating that the overexpression of KLK6 reversed the inhibitory effects of miR-431-5p mimics on cell migration and invasion. This suggests that miR-431-5p regulates cell migration and invasion by targeting KLK6, thereby mediating the occurrence and development of CRC.

## Discussion

CRC is a highly invasive cancer of the digestive tract [6]. Since metastasis may occur early, most patients have a poor prognosis, leading to a rapid decline in their quality of life [25]. As society and economy advance rapidly, the aging of the population, and the change of residents’ lifestyle and diet structure, the incidence and fatality rate of CRC in China are increasing year by year and showing a trend of younger age [26]. Therefore, how to better achieve early screening and diagnosis is of great significance for CRC. Cancer suppressor gene or oncogene are one of the hotspots in CRC research at present [8].

In this study, based on the GEO and KEGG database, 39 significantly differentially expressed miRNAs related to CRC were screened, among which 19 were up-regulated and 20 were down-regulated; there were 3716 significant differentially DEGs were identified, with 1839 down-regulated and 1877 up-regulated. GO enrichment analysis showed that the DEGs were primarily enriched in immunoglobulin complex, external side of plasma membrane, and synaptic membrane; KEGG enrichment pathways included Wnt signaling pathway, cAMP signaling pathway and neuroactive ligand-receptor interaction. Abnormal activation or overexpression of Wnt could lead to β-catenin accumulation in both nucleus and cytoplasm, inducing the transcription of Wnt downstream target genes, and then promoting the malignant proliferation and early carcinogenesis of colon cells [27, 28].

WGCNA could use expression patterns to classify genes and explore the association of candidate modules with clinical factors, explaining the biological significance of gene modules and mining prognostic genes from a biological point of view [29]. In this study, DEGs were analyzed by WGCNA, and it was found that the modules black and magenta showed a strong positive correlation with tumor tissue. Both GO and KEGG enrichment analyses of the module genes revealed that primarily enriched pathways included signaling receptor activator activity, receptor ligand activity, keratinization, ECM-receptor interaction, Cytokine-cytokine receptor interaction, JAK-STAT signaling pathway and Wnt signaling pathway. The hub genes screened by PPI included KRT16, KLK6, KRT6C, KRT6A, etc. Among them, patients with high KLK6 expression had a worse prognosis than those with low KLK6 expression, indicating that high expression of the KLK6 gene promotes the development of CRC. Previous studies have reported that an increase in KLK6 expression is associated with a poor prognosis in CRC patients [30]. Moreover, existing research has shown that CRC patients with low KLK6 expression have a higher survival rate and a better prognosis, while those with high expression have the opposite outcome [31]. This finding was replicated in the current study.

Kallikrein6 (KLK6) gene is a member of KLKs family and encodes a human kallikrein 6 (hK6) protein consisting of 223 amino acids with trypsin-like activity [32]. KLKs are intimately correlated to tumor occurrence, development, invasion and metastasis, being essential in the tumor microenvironment [33]. Some KLKs have been used in clinical practice as tumor markers [34–36]. For example, KLK3 has been widely used as a marker of prostate cancer in clinical screening, diagnosis, prognosis and monitoring [32]. KLK13 is reported as an independent and favorable prognostic marker in breast cancer and ovarian cancer [37]. KLK6, KLK7, KLK8 and KLK10 are considered as excellent biomarker candidates in diagnosing colon adenocarcinoma [38]. KLK6 is considered to be associated with proliferation, adhesion, and malignant transformation of tumor tissue, being an important factor responsible for poor prognosis [39]. Studies on KLK6 and CRC have revealed significantly high KLK6 expression in CRC tissues as compared to normal tissues, which is associated with tumor serosa infiltration, liver metastasis, clinical stage, and poor prognosis [40]. Kim et al. [41] have unveiled that KLK6 expression in the tissues and serum of CRC patients increases markedly, which is responsible for poor disease prognosis. Knockdown of KLK6 in colon cancer cells inhibits migration and invasion in vitro [42]. However, the mechanism of KLK6 in CRC is still elusive. In this study, it was found that miR-431-5p could target the KLK6 gene through targeting verification, which could be a therapeutic target.

MiRNAs are important in the genesis, development and treatment of tumors, which can play as either tumor promoters or tumor suppressors [9, 43–46]. MiRNA in CRC tissue cells is either down-regulated or up-regulated to varying degrees, and it participates in the occurrence and development of CRC through the interaction with proto-oncogene, tumor suppressor gene and other cancer-related genes, which may be related with treatment sensitivity [47]. MiR-431-5p has been reported to have an antitumor role in multiple cancers [48]. MiR-431-5p inhibits epithelial mesenchymal transformation of hepatoma cells by down-regulating the expression of UROC28 [12]. MiR-431-5p may mediate the growth of thyroid papillary cells by directly binding and reducing the expression of CDK14 [49]. Studies have reported that miR-431-5p is down-regulated in CRC and is associated with poor prognosis [50]. However, there are few reports on the targeting of KLK6 by miR-431-5p. This study predicted KLK6 related to CRC through bioinformatics analysis and found that miR-431-5p might target KLK6 through target relationship prediction. Our cell experiments revealed that miR-431-5p could regulate the expression of KLK6. Dual luciferase reporter assays and cell rescue experiments demonstrated that miR-431-5p targeted KLK6, thereby regulating the occurrence and development of CRC. These results suggest that miR-431-5p holds significant potential in the treatment of CRC by regulating KLK6. It is expected to become a new targeted treatment strategy, inhibiting the expression of KLK6 to halt the progression of the disease. In future research, we can explore the use of small-molecule compounds or miR-431-5p mimics to regulate KLK6, opening up new avenues for the treatment of CRC. In addition, repurposing drugs and administering various vitamins, such as E and D, as prophylactics may have modulatory effects and positive impacts on CRC [51].

In this study, genes related to CRC and their molecular mechanisms were revealed through various bioinformatics methods. The research found that miR-431-5p targets KLK6 and regulates its expression, which provides potential biomarkers and targeted treatment strategies for the clinical diagnosis and treatment of CRC. Nevertheless, this study also has some limitations. The experiments were performed only at the cellular level, and further validation in animal models is necessary as well as in clinical settings in the future.

## Conclusion

The target gene prediction of the bioinformatics analysis done in this study showed that miR-431-5p could target the hub gene, KLK6. This was confirmed by cellular validation. The results suggest that miR-431-5p suppresses the role of KLK6 in the development of CRC. As such, the use of small molecules to inhibit KLK6 through miR-431-5p offers a promising approach to CRC.

## Data Availability

All data generated or analysed during this study are included in this published article.

## Abbreviations

miR: microRNA
CRC: Colorectal cancer
KLK6: Kallikrein-related peptidase 6
GEO: Gene Expression Omnibus
TCGA: The Cancer Genome Atlas
WGCNA: Weighted Gene Co-expression Network Analysis
EGFR: Epidermal growth factor receptor
PANX3: Pannexin 3
GEPIA: Gene Expression Profiling Interactive Analysis
DAVID: Database for Annotation, Visualization and Integrated Discovery
GO: Gene Ontology
KEGG: Kyoto Encyclopedia of Genes and Genomes
WGCNA: Weighted Gene Co-Expression Network Analysis
TOM: Topological Overlap Matrix
PPI: Protein-protein Interaction
STRING: Search Tool for the Retrieval of Interacting Genes/Proteins
MCC: Matthews Correlation Coefficient
OS: Overall Survival
MEM: Minimum Essential Medium
FBS: Fetal Bovine Serum
RIPA: Radio-Immunoprecipitation Assay Buffer
SDS: Sodium Dodecyl Sulfate
ECL: Enhanced Chemiluminescence
BP: Biological Process
CC: Cellular Component
MF: Molecular Function
KLK6: Kallikrein6
hK6: Human kallikrein 6
UROC28: Prostate and Breast Cancer Overexpressed 1
CDK14: Cyclin-Dependent Kinase 14
